# Skeletal Muscle Perilipin 3 and Coatomer Proteins Are Increased following Exercise and Are Associated with Fat Oxidation

**DOI:** 10.1371/journal.pone.0091675

**Published:** 2014-03-14

**Authors:** Jeffrey D. Covington, Jose E. Galgani, Cedric Moro, Jamie M. LaGrange, Zhengyu Zhang, Arild C. Rustan, Eric Ravussin, Sudip Bajpeyi

**Affiliations:** 1 Pennington Biomedical Research Center, Baton Rouge, Louisiana, United States of America; 2 School of Medicine, Pontificia Universidad Catolica de Chile, Santiago, Chile; 3 Inserm UMR 1048, Institute of Metabolic and Cardiovascular Diseases and Paul Sabatier University, Toulouse, France; 4 Department of Pharmaceutical Biosciences, University of Oslo, Oslo, Norway; 5 Department of Kinesiology, University of Texas in El Paso, El Paso, Texas, United States of America; Clermont Université, France

## Abstract

Lipid droplet-associated proteins such as perilipin 3 (PLIN3) and coatomer GTPase proteins (GBF1, ARF1, Sec23a, and ARFRP1) are expressed in skeletal muscle but little is known so far as to their regulation of lipolysis. We aimed here to explore the effects of lipolytic stimulation *in vitro* in primary human myotubes as well as *in vivo* following an acute exercise bout. *In vitro* lipolytic stimulation by epinephrine (100 μM) or by a lipolytic cocktail (30 μM palmitate, 4 μM forskolin, and 0.5 μM ionomycin, PFI) resulted in increases in PLIN3 protein content. Coatomer GTPases such as GBF1, ARF1, Sec23a, and ARFRP1 also increased in response to lipolytic stimuli. Furthermore, a long duration endurance exercise bout (20 males; age 24.0±4.5 y; BMI 23.6±1.8 kg/m^2^) increased PLIN3 protein in human skeletal muscle (p = 0.03) in proportion to *ex vivo* palmitate oxidation (r = 0.45, p = 0.04) and whole body *in vivo* fat oxidation (r = 0.52, p = 0.03). Protein content of ARF1 was increased (p = 0.04) while mRNA expression was increased for several other coatomers (GBF1, ARF1, and Sec23a, all p<0.05). These data provide novel observational insight into the possible relationships between lipolysis and PLIN3 along with these coatomoer GTPase proteins in human skeletal muscle.

## Introduction

Ever since the discovery that intramyocellular lipid (IMCL) correlated inversely with insulin sensitivity in skeletal muscle, understanding lipid metabolism in skeletal muscle has been at the cusp of insulin resistance research. Lipid droplet dynamics is an integral part to understanding overall lipid metabolism. [1]–[3]. Paralleled to that, it has also been shown that endurance trained athletes as well as individuals with type 2 diabetes both have elevated IMCL, while the insulin sensitivities in these two groups remain polar opposites, [4] thus, indicating that merely having elevated IMCL does not necessarily result in insulin resistance. Therefore, understanding the regulation of lipid droplet dynamics within skeletal muscle would likely be an integral part to understanding overall lipid metabolism. Studies into the perilipin family of proteins have demonstrated that perilipin 2 (PLIN2, also known as ADRP) and perilipin 5 (PLIN5, also known as OXPAT) appear to be fundamental to lipid droplet oxidation and interaction of lipid droplets to mitochondria [5]–[12]. However, few investigators have explored the regulation of perilipin 3 (PLIN3, also known as TIP47), a prominent, highly expressed lipid droplet-associated protein in skeletal muscle and its impact on lipid oxidation [12]–[14].

PLIN3 co-localizes to lipid droplets upon epinephrine stimulation and contraction in rat skeletal muscle [15]. Likewise, adipose triglyceride lipase (ATGL), a major lipase present in skeletal muscle, has been shown to co-localize to PLIN3 coated lipid droplets [14], [16]. Coatomer GTPases may have a possible regulatory role in the delivery of ATGL to PLIN3-coated lipid droplets via transport through the endoplasmic reticulum to Golgi apparatus [17]. Treatment with brefeldin A, a compound known to inhibit the ER-to-Golgi transport, prevents ATGL from localizing to PLIN3-coated lipid droplets in HeLa cells [17]. Furthermore, knockdown of ADP-ribosylation factor 1 (ARF1), ARF related protein 1 (ARFRP1), and Golgi-specific brefeldin A-resistance guanine nucleotide exchange factor 1 (GBF1) in HeLa cells and brown adipose tissue also prevents the co-localization of ATGL to PLIN3 coated lipid droplets [17], [18]. To date, only a few studies have investigated these pathways in skeletal muscle or in relation to exercise stimulation and therefore lipid availability for oxidative metabolism [13], [14]. Since endurance exercise is heavily dependent on lipid oxidation [19] and that endurance exercise trained athletes have high levels of IMCL [4], we hypothesized that perilipin 3 would be associated with exercise-induced lipolysis.

We therefore investigated the effect of exercise on PLIN3 protein and lipid droplet associated coatomers using both *in vitro* and *in vivo* approaches. *In vitro* experiments were performed using epinephrine [20] to stimulate lipolysis as well as a pharmacological cocktail of palmitate, forskolin, and ionomycin (PFI) to induce lipolysis in a primary human skeletal muscle cell culture model [21]. PLIN3 was also measured from human skeletal muscle biopsies taken before and after a long-duration endurance exercise bout. Furthermore, we investigated the expression of ATGL and the GTPases known to regulate ER-to-Golgi trafficking. These data suggest an important role of PLIN3 and ER-to-Golgi coatomers in relation to skeletal muscle lipid metabolism, and offers insight into potentially new lipolytic pathways for lipid metabolism in skeletal muscle.

## Methods

### Ethics Statement

The participants provided their written informed consent and all aspects of the study protocol were reviewed and approved by the Institutional Review Board at Pennington Biomedical Research Center.

### Establishment of Primary Human Skeletal Muscle Cultures

Primary muscle cultures were established from muscle biopsies obtained from the *vastus lateralis* in five lean, healthy Caucasian male donors (Age 23.0±1.9 yrs and BMI 24.2±0.6 kg/m^2^). Establishment of human primary muscle culture has been modified from protocols as previously described [22]. Myoblast skeletal muscle progenitor cells were immuno-sorted using the 5.1H11 antibody provided by the Hybridoma Bank (University of Iowa) and the MACS cell sorting column system (Miltenyi Biotec, Auburn, CA). Myoblasts cultures from the five donors were grown simultaneously to approximately 90% confluence and then pooled together for experiments using protocols previously described [23]. Cells were further grown to approximately 80% confluence, and then treated with α-Minimum Essential Medium (Life Technologies, Grand Island, NY) supplemented with 2% fetal bovine serum (Life Technologies, Grand Island, NY), 1% bovine fetuin (Sigma, St. Louis, MO), and 1% Penstrep at 5 mg/mL (Life Technologies, Grand Island, NY) to trigger differentiation. Cells were maintained in differentiation media for 7 days and were considered myotubes upon visual assessment of fused, longitudinal, multinucleated cells.

### *In vitro* Epinephrine and PFI Treatment with Primary Human Myotubes

Myotubes were treated using techniques adapted from Watt et al. [20] with 100 μM Epinephrine (Sigma, St. Louis, MO) for a time course of 15 minutes, 30 minutes, and 1 hour with collection of total protein at each time point. For PFI experiments, myotubes were treated with 30 μM palmitate, 4 μM forskolin, and 0.5 μM ionomycin (PFI) – all purchased from Sigma (St. Louis, MO). We previously showed that PFI-treatment in myotubes increased palmitate oxidation, increased mitochondrial oxidative phosphorylation complexes expression, and improved glucose uptake [21]. Briefly, myotubes were maintained in differentiation media for 4 days and then treated for 1 hour each day with PFI for 3 additional days. Differentiation media was similarly changed each day, without PFI, for control cells. Following 3 days of PFI treatment, total protein and mRNA were collected immediately following PFI (0 minutes) and for a time course of 15 minutes, 30 minutes, and 1 hour after PFI treatment. Total protein was collected using RIPA buffer (Sigma, St. Louis, MO) supplemented with 2% Protease Inhibitor Cocktail (Sigma, St. Louis, MO), 2% Phosphatase Inhibitor Cocktail 2 (Sigma, St. Louis, MO), and 2% Phosphatase Inhibitor Cocktail 3 (Sigma, St. Louis, MO). Total mRNA was collected using QIAzol (Qiagen, Germantown, MD).

### Endurance Exercise Bout in Human Participants

Twenty healthy, normoglycemic male participants (16 Caucasian, 3 African American, 1 non-specified race) who were not engaged in sports at competitive level, were recruited to participate in this trial. Characteristics of these participants are provided in Table 1. Body composition was assessed by dual x-ray absorptiometry (DXA, QDR 4500A; Hologics, Waltham, MA) and VO_2_max was measured on a stationary bicycle ergometer (Lode Excalibur, Groningen, The Netherlands) using an incremental workload protocol with simultaneous gas exchange measurements using a metabolic cart (TrueOne 2400; ParvoMedics, Sandy, UT). VO_2_max and DXA measurements were assessed at a period of greater than 2 days before the exercise intervention in order to prevent any confounding acute effects of exercise on baseline measurements. Before the exercise bout, participants were admitted in the evening to the institutional in-patient unit. The following morning, after an overnight fast, resting metabolic rate was measured using a DeltaTrac metabolic cart and a percutaneous skeletal muscle biopsy of the *vastus lateralis* muscle was performed. Additional results of this exercise trial have already been published [24]. Gas exchange while exercising was assessed from expired air collected by mouthpiece using a Parvomedics TrueOne 2400 metabolic cart. Total energy expenditure and substrate oxidation were calculated as previously described [25]. Participants then exercised on a stationary bike at 50% their VO_2_max until they had expended 650 kcals. Indirect calorimetry measures were performed after 8%, 20%, 40%, 60%, 80% and right before exercise completion in order to gage when 650 kcals of energy had been expended. Blood was drawn at regular intervals coupled to indirect calorimetry measures with epinephrine and norepinephrine determined by chemiluminescent immunoassay (Immulite 2000™, Siemens Healthcare Diagnostics, Deerfield, IL); serum glucose, insulin, and lipids by an enzymatic assay on a Beckman Coulter DXC 600 (Beckman Coulter, Brea, CA). Manufacturer's protocols were followed for all the serum measurements. Here we have only reported serum measures from before and after exercise (Table 2). Immediately following the exercise bout, a second percutaneous skeletal muscle biopsy was obtained from the *vastus lateralis* proximal to the first biopsy.

**Table 1:** Anthropometric Characteristics of Participants in the Single Endurance Exercise Bout.

	Mean ± SD
**Age (Yr)**	24.0±4.5
**Weight (kg)**	76.7±6.5
**Height (cm)**	180.3±5.4
**BMI (kg/m^2^)**	23.6±1.8
**% Body Fat**	16.6±3.2
**FM (kg)**	12.8±3.1
**FFM (kg)**	63.9±4.7
**VO_2Max_ (mL/min/FFM)**	47.2±5.7
**Fasting Glucose (mg/dL)**	88.0±4.6
**Fasting Insulin (mU/dL)**	3.6±1.6
**HOMA-IR**	0.78±0.37
**Fasting FFA**	0.45±0.17
**% of Type 1 fibers**	35.9±11.7
**% of Type 2 fibers**	64.2±11.7

**Table 2:** Clinical and Muscle Fiber Characteristics Before and After the Single Endurance Exercise Bout.

	Baseline	Post-Exercise	
	Mean ± SD	Mean ± SD	p value
**RER**	0.95±0.04	0.89±0.03	<0.001
**Palmitate Oxidation, ** ***ex vivo*** ** (nmol/hr/mg protein)**	615.9±375.9	887.3±404.3	0.01
**Total IMCL Content (AU)**	27.7±27.5	21.3±19.4	0.21
**IMCL Content in type 1 fibers (AU)**	29.3±28.6	23.4±22.6	0.21
**IMCL Content in type 2 fibers (AU)**	25.9±25.9	20.1±17.9	0.25
**IMCL Fiber type 1 density (AU)**	11.2±13.6	7.9±7.5	0.24
**IMCL Fiber type 2 density (AU)**	16.3±16.9	13.3±13.3	0.40
**Glycogen Content (AU)**	8.40±0.79	7.32±0.68	0.001
**FFA (mmol/L)**	0.45±0.17	0.73±0.30	<0.001
**Epinephrine (mg/dL)**	46.9±17.8	193.4±77.5	<0.001
**Norepinephrine (mg/dL)**	302.2±149.0	986.2±347.6	<0.001

### Skeletal Muscle Biopsy Procedure

After local anesthesia with lidocaine/bupivacaine, skeletal muscle samples were collected using the Bergstrom technique with suction from the *vastus lateralis* (Propper Manufacturing Co., Long Island City, NY). Two separate incisions were made to collect tissues at baseline and post-exercise. The second biopsy collections were obtained within a time frame of no more than 3 minutes following the completion of exercise. All skeletal muscle samples were visually assessed and cleared for intramuscular adipose tissue, and then immediately snap frozen in liquid nitrogen for mRNA and Protein measures. Samples were blotted dry and then mounted in a mixture of Optimal Cutting Temperature (OCT, Thermo Scientific, Waltham, MA) and Tragacanth powder (Acros, Geel, Belgium) for immunohistochemical measures of glycogen, intramyocellular lipid, and fiber typing. Another sample was collected for measurements of *ex vivo* palmitate oxidation.

### Immunohistochemical Measures

Measures of fiber typing and intramyocellular lipid (IMCL) were performed as previously described using immunofluorescence techniques [24]. Fiber typing was done by immunohistochemistry performed on 12 μm sections, obtained by using a Microm HM 550 (Thermo Scientific, Waltham, MA). A mouse monoclonal antibody specific for slow myosin heavy chain type 1 (MHC1) was used to detect type 1 fibers (MAB1628; Millipore, Burlington, MA), and a rat monoclonal antibody against laminin (AB2500; Abcam Inc, Cambridge, MA) was used to detect myofiber cell membranes. Sections were then counterstained with Bodipy 494 dye (Molecular Probes, Eugene, OR) to stain for IMCL. Images were taken using a confocal microscope (Leica TCS SP5 AOBS resonant scanning multiphoton confocal microscope, Leica Microsystems, Wetzlar, Germany) and type I fibers were counted. IMCL was determined using the Sigma Scan Pro 5 software (SPSS, Chicago, IL) by delineating Bodipy staining within myofibers. Glycogen content was measured using Periodic Acid, Shiff staining and analyzed using the Sigma Scan Pro 5 software [26]. Representative images from before and after the exercise intervention for IMCL, fiber typing, and glycogen are provided in figure S2. For all histology measures, three cross-sectional slices were obtained within the tissue. No less than 50 fibers were assessed from each cross-sectional slice for IMCL content, fiber type, and glycogen.

### *Ex vivo* Palmitate Oxidation Measures in Skeletal Muscle

The palmitate oxidation assay was performed in skeletal muscle as previously described [24]. Briefly, approximately 75 mg of skeletal muscle tissue was homogenized and loaded into a trapping plate apparatus to assess gas exchange for fatty acid oxidation. 0.176 μM of total palmitate (0.088 μM of [1-^14^C]-palmitate in 0.088 μM of non-radiolabeled palmitate) was added to the muscle homogenate. Radiolabeled palmitate was obtained from American Radiolabeled Chemicals (St. Louis, MO). Radiolabeled ^14^CO_2_ and incomplete acid soluble intermediates from palmitate oxidation were assessed using scintillation counting. Data was adjusted to total protein content obtained from muscle homogenate as determined through the bicinchoninic acid assay (Pierce BCA, Thermo Scientific, Waltham, MA).

### Gene Expression in Skeletal Muscle

Total mRNA from both *in vivo* and *in vitro* experiments was extracted using the miRNEasy Mini Kit (Qiagen, Germantown, MD), and cDNA was made using the High Capacity cDNA Kit (Applied Biosystems, Foster City, CA). Detection of gene expression was performed using TaqMan Gene Expression Assays-on-Demand (Applied Biosystems, Foster City, CA); a list of catalogue numbers for each gene product is provided in Table S1. Real-Time qPCR was carried out using the 7900HT Fast Real-Time PCR system (Applied Biosystems, Foster City, CA), and expression levels were determined against a standard curve. Skeletal muscle and myotube gene expression was adjusted to the expression of RPLPO.

### Protein Content in Skeletal Muscle

Protein content was measured from total protein extracts by western immunoblotting using the Criterion apparatus and 12.5% SDS-polyacrylamide gels (all purchased from Bio-Rad, Hercules, CA) and adjusted to either GAPDH (AB9484; AbCam, Cambridge, MA) or total protein assessed by Ponceau S stain (Sigma, St. Louis, MO). The antibody for PLIN3 was purchased from Novus Biologicals (Cat no. NB110-40764, Littleton, CO). The antibodies for GBF1 (AB86071), ATGL (AB109251), and ARFRP1 (AB108199) were purchased from AbCam (Cambridge, MA). The antibody for ARF1 was purchased from Epitomics (Cat no. 1635-1; Burlingame, CA).

### Statistical Analysis

Data was analyzed using PRISM GraphPad Software, version 6.0 (GraphPad Software, La Jolla, CA). All data were found to be normally distributed using the Shapiro-Wilk Normality test. A paired, Student two-way t-test was used to assess baseline and intervention measures, and Pearson's correlations were used in the exercise intervention. A one-way ANOVA was used to determine differences in gene expressions at different time points for the *in vitro* PFI experiments. A p value<0.05 was considered statistically significant.

## Results

### PLIN3 changes in response to *in vitro* epinephrine and lipolytic cocktail (PFI treatment) in human myotubes

**Figure 1:**
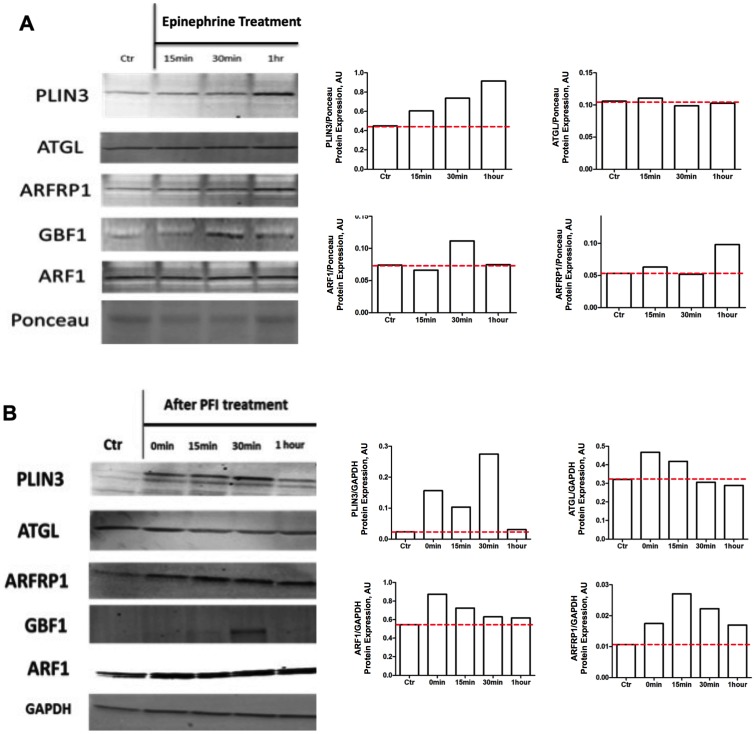
Epinephrine and lipolytic cocktail (PFI) in human primary myotubes increases perilipin 3 and coatomer proteins. A) Primary human skeletal muscle myotubes (n = 5) were stimulated with 100 uM of epinephrine. PLIN3 expression as assessed by densitometry increased continuously with epinephrine. Additionally, GTPases ARF1 and GBF1 increased 30 minutes following epinephrine stimulation and ARFRP1 increased 1 hour following epinephrine stimulation. B) PLIN3, ATGL ARFRP1, GBF1 and ARF1 protein content increased after *in vitro* lipolytic stimulus (PFI) in primary human skeletal muscle myotube (n = 5).

The PLIN3 protein content increased steadily with epinephrine stimulation throughout the time course (Figure 1A). Likewise, the ER-to-Golgi coatomer GTPases ARF1 and ARFRP1 also increased (Figure 1A) 30 minutes and 1 hour respectively following the epinephrine stimulation. The coatomer GTPase, GBF1 was maximally expressed after 30 minutes of epinephrine prior to the noticeable maximal increases in PLIN3 and ARFRP1 (Figure 1A). ATGL levels were virtually not changed during the duration of epinephrine treatment (Figure 1A).

In response to the lipolytic cocktail (PFI), PLIN3 protein content increased immediately following PFI treatment when compared to control conditions, achieving maximal values after 30 minutes (Figure 1B). Similarly, the coatomer GTPase GBF1 had maximal expression that coincided with PLIN3 maximal expression at the 30 minute time point (Figure 1B). PFI treatment increased the expression of the coatomer GTPase ARFRP1 (Figure 1B). Finally, ATGL expression was increased, albeit slightly, over controls immediately upon the completion of PFI treatment that steadily declined up to 1 hour after PFI treatment (Figure 1B).

**Figure 2:**
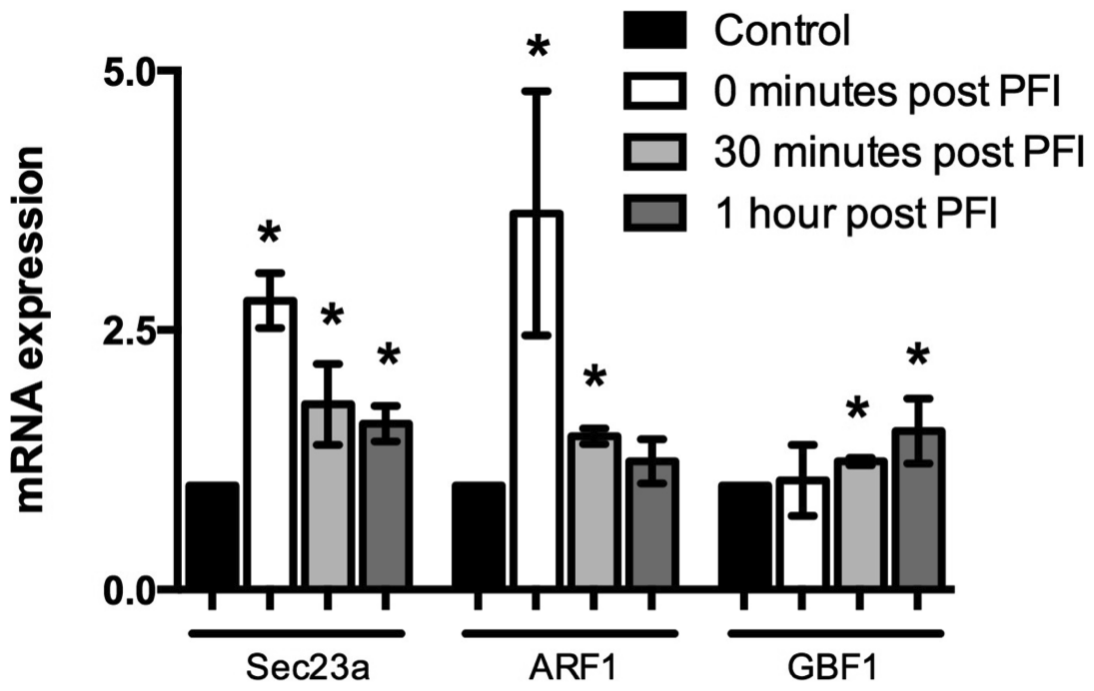
Coatomer GTPase gene expression changes with lipolytic cocktail (PFI) in human primary myotubes. mRNA level of Sec23a, ARF1 and GBF1 in cultured human myotubes before and after PFI treatment (0, 30 min and 1 h) (n = 5). *p<0.05

Regarding mRNA expression levels, *sec23a*, a subunit of the coatomer 2 protein complex (COPII), and *arf1* increased 2.5 and 3-fold respectively immediately after PFI treatment (Figure 2). The mRNA expression of *gbf1* did not reach significant increase until 30 minutes and 1 hour following PFI treatment (Figure 2).

### PLIN3 changes in response to an endurance exercise bout in human muscle

**Figure 3:**
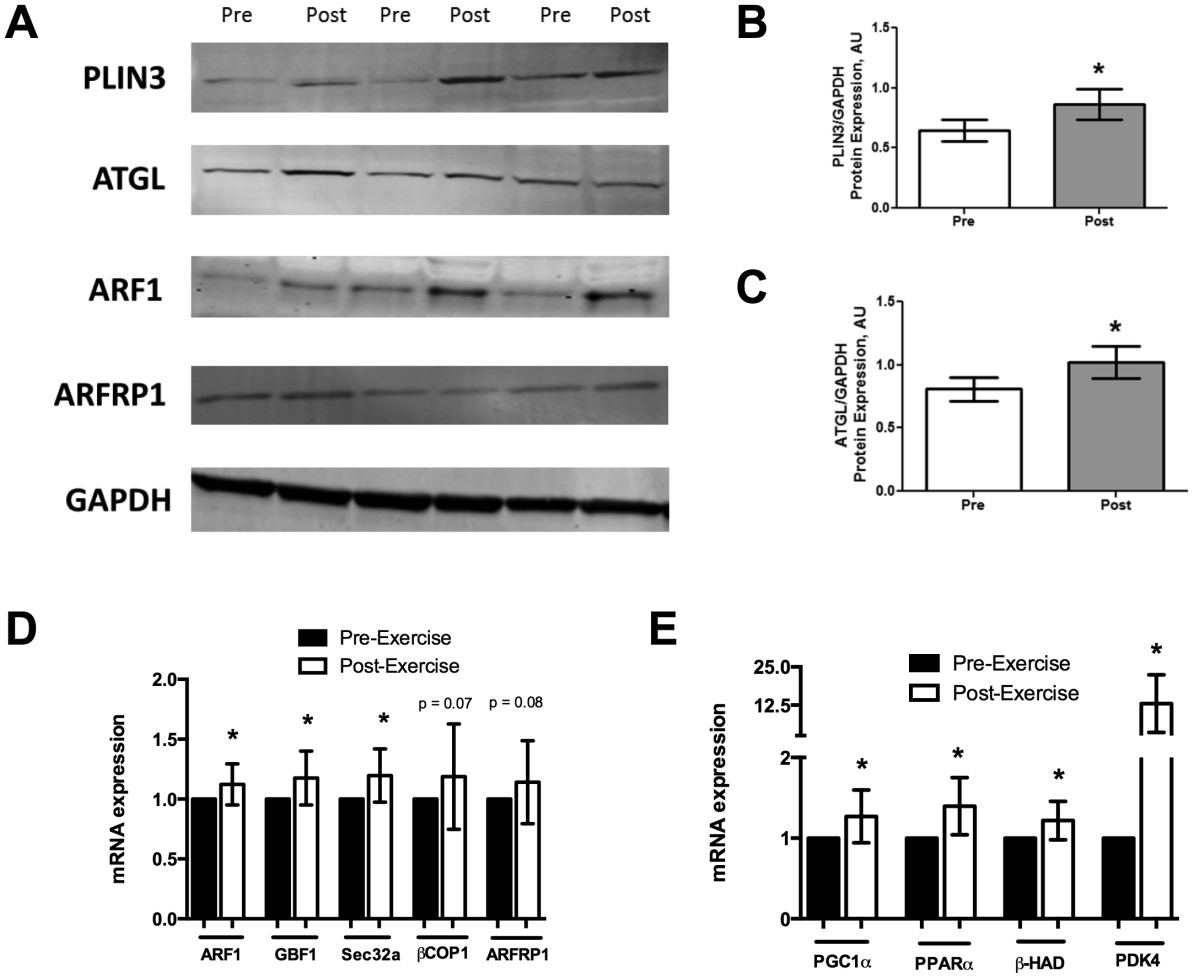
Changes in protein and gene expression relating to lipolysis with a single bout of endurance exercise in human skeletal muscle. A) Representative blots of PLIN3, ATGL, ARF1, ARFRP1 and loading control GAPDH. B) Quantitative bar graph of skeletal muscle PLIN3 protein after an acute exercise bout (n = 19). C) Quantitative bar graph of skeletal muscle ATGL protein after an acute exercise bout (n = 19). D) mRNA levels of lipid droplet coatomer genes (n = 19), and (E) mRNA levels of oxidative genes, in skeletal muscle of healthy subjects in response to an acute exercise bout (n = 14–19). *p<0.05.

The endurance exercise bout was successful at reducing whole-body respiratory exchange ratio (RER), increasing serum free fatty acid (FFA) concentration and *ex vivo* muscle palmitate oxidation, thus confirming that lipid oxidation was being favored over carbohydrate oxidation (Table 2). In parallel to the increased lipid oxidation, PLIN3 and ATGL protein content increased with exercise (Figure 3A, 3B and 3C). Similarly, the coatomer GTPase ARF1 increased at both protein and the mRNA level (Figure 3A and 3D); *arfrp1*, *gbf1*, *sec23a*, and *βcop1* increased only at the mRNA level (Figure 3D). Both *pparα*, a transcription factor known to regulate the expression of the perilipin protein family [27]-[29], and its co-activator *pgc1α* increased their mRNA expression with exercise. mRNA expression of *βhad* and *pdk4*, two enzymes that favors fat oxidation, were also increased with exercise, further confirming lipid oxidation being favored in the skeletal muscle with exercise (Figure 3E).

**Figure 4:**
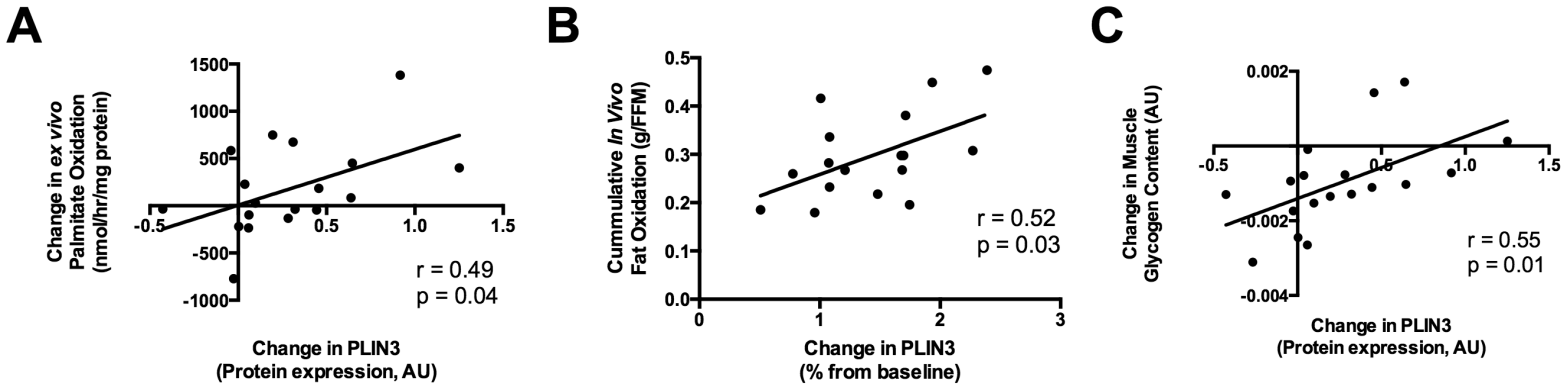
Associations between change in perilipin 3 (PLIN3) protein in skeletal muscle tissue and fat oxidation. A) Correlation between the change in PLIN3 protein expression and the change in *ex vivo* palmitate oxidation measured from skeletal muscle tissue homogenates (n = 18). B) Correlation between the percent change in PLIN3 protein expression and whole body cumulative fat oxidation measured by indirect calorimetry adjusted for fat free mass (n = 18). C) Correlation between the change in total glycogen content and the change in PLIN3 protein during exercise (n = 18).

Changes in PLIN3 protein with exercise were positively associated with both the change in *ex vivo* muscle palmitate oxidation (r = 0.49, p = 0.04; Figure 4A) and with cumulative whole-body fat oxidation after adjusting for fat-free mass (FFM; r = 0.52, p = 0.03; Figure 4B). We noted a slight decrease in IMCL content; however, despite increases in *in vivo* and *ex vivo* fat oxidations, the decrease in IMCL content did not reach significance (p = 0.21, Table 2). We found that those individuals who increased PLIN3 with exercise tended to have an inverse relationship to the change in IMCL (r = −0.35, p = 0.10; Figure S1A). However, after adjusting for fiber-type cross-sectional area, a measure referred to as IMCL density, participants who increased PLIN3 protein content with exercise had a significant inverse correlation with IMCL density in type II fibers (r = −0.58, p = 0.02, Figure S1B); whereas there was no relation with IMCL density in type I fibers (Figure S1C). As expected, glycogen content in the skeletal muscle significantly decreased after exercise (p = 0.001, Table 2). Changes in PLIN3 protein content were positively associated with changes in glycogen (r = −0.55, p = 0.01; Figure 4C), suggesting that those participants who increased their PLIN3 favored lipid oxidation over carbohydrate oxidation during exercise. Changes in PLIN3 protein, however, did not have any relation with changes in serum FFA, epinephrine or norepinephrine concentrations.

## Discussion

Perilipin 3 is a lipid droplet coat protein previously shown to co-localize to lipid droplets and with ATGL upon lipolytic stimulation [14], [16]. Our data shows for the first time in human primary muscle cells that PLIN3 expression increases in response to both epinephrine stimulation and to a pharmacological cocktail known to induce lipolysis. Importantly, we also show for the first time that PLIN3 expression increases *in* vivo, in skeletal muscle tissue of healthy, lean males following a long endurance exercise bout. The increase in PLIN3 protein correlated positively with *ex vivo* muscle homogenate palmitate oxidation as well as whole-body cumulative fat oxidation with exercise.

Our data supports the hypothesis that PLIN3 is involved in lipid oxidation within skeletal muscle. Prats et al. showed that in individual rat muscle fibers, PLIN3 co-localizes to lipid droplets after epinephrine administration [15]. Smirnova et al. showed that during lipolysis, ATGL co-localizes to lipid droplets coated with PLIN3 and replaces PLIN3 along the surface of lipid droplets [16]. Our PFI treatment in human myotubes demonstrates that PLIN3 is increased immediately after the cessation of the lipolytic stimulus (Figure 1B). Additionally, PLIN3 expression increases from immediately following PFI treatment up to 30 minutes, while maximal expression of ATGL is immediately following PFI treatment with a steady decline in its expression following the lipolytic stimulation (Figure 1B). This supports the concept purported in Smirnova et al. stating that ATGL replaces PLIN3 during lipolysis. Furthermore, we show that PLIN3 in myotubes increases in response to *in vitro* epinephrine stimulation as well as *in vivo* after an endurance exercise bout (Figure 1A, 3A, and 3B). The increases in PLIN3 protein content in tissue were positively associated with changes in *ex vivo* palmitate oxidation in skeletal muscle (Figure 4A), cumulative whole-body fat oxidation (Figure 4B), and with changes in glycogen content in the muscle (Figure 4C). Those individuals who increased their PLIN3 protein content with exercise, had a trend towards an inverse relation to changes in IMCL content with exercise (Figure S1). Together, these data suggest that PLIN3 protein is involved in lipolysis induced by either *in vitro* pharmacological stimuli or by endurance exercise in the skeletal muscle.

Our data also shows increases in several coatomer GTPases after lipolytic stimulation both *in vitro* and *in vivo*. Previous experiments have shown that knockdown of βCOP1, ARF1, GBF1, or Sec23a prevents the colocalization of ATGL to PLIN3 coated lipid droplets [17]. Guo et al. hypothesized that the coatomer GTPases are involved in the partitioning of lipid droplets for lipolysis and lipase interactions [30]. However, no direct protein-protein interaction between ATGL and PLIN3 has been observed [31]. Therefore, we speculate that an increase in PLIN3 along with coatomers GTPases suggests that PLIN3 facilitates lipolysis by serving as a targeting signal for directing lipase delivery to lipid droplets. Our data reported here, though is novel, is observational and a true mechanism of these relationships cannot be fully elucidated from this study. Future proteomic investigations will be necessary to conclusively extrapolate a definitive mechanism for their interaction.

One of the limitations of our study is that although we recruited 20 male participants, we were only able to perform molecular investigations in 14 to 19 participants for some of our measures, due to limited amounts of skeletal muscle tissue. Additionally, since we collected biopsy samples at baseline and immediately after exercise bout, we are only able to provide the acute effect of exercise on PLIN3 content. An inverse correlation between changes in PLIN3 and IMCL density in type II fibers was observed but cannot be explained based on our current data. Future studies on fiber type specific IMCL and PLIN3 expression may provide insight to this correlation. Furthermore, based on these observational studies we cannot provide insight as to why the maximal expression of mRNA and protein level of GTPases investigated occur at different time points following the lipolytic stimulation (Figure 1A and 1B). We can merely state that increases in GBF1, ARFRP1, ARF1, Sec23a, and βCOP1 occur either at the mRNA level, the protein level or both at some time points following lipolytic stimulation. However, it is important to note that this is the first study that demonstrates an increase in PLIN3 and coatomer GTPases in human skeletal muscle following lipolytic stimulation both using *in vitro* and *in vivo* experiments. Future studies would be necessary to determine the effects of lipolytic stimulus duration, signal transduction, co-localization, and protein-protein interactions between PLIN3 and coatomer GTPases in skeletal muscle lipid metabolism. Interestingly, Louche et al. recently reported an upregulation of PLIN3 protein content after 8 weeks of endurance exercise training [32]. Future studies should also be conducted to determine the chronic effects of exercise and various types of exercise training on the PLIN3 in relation to lipid oxidation.

In conclusion, our data demonstrates for the first time that the increase in expression of perilipin 3 and coatomer associated targets involved in ER-to-Golgi cargo transport with exercise are involved with exercise-stimulated lipolysis in skeletal muscle. These data offer a previously unexplored potential pathway for the regulation of lipase access to nascent or fragmenting lipid droplets and the regulation of lipolysis within skeletal muscle. This may provide further insight into potential aberrations in skeletal muscle fat oxidation, which is shown to occur in obesity, type 2 diabetes and lipodystrophy.

## Supporting Information

Figure S1 **The change in PLIN3 protein content did not significantly correlate with the changes in IMCL with exercise in all participants.** A) Nevertheless, for those participants who increased their PLIN3 protein content had a trend to decrease their IMCL content with exercise (p = 0.1). B) Furthermore, the change in IMCL density in type II fibers inversely related to those participants who increased their PLIN3 expression with exercise (p = 0.02). C) However, there was no significance association between the change in PLIN3 expression and the change in IMCL density in type I fibers.
(TIFF)

Figure S2 **Representative images of intramyocellular lipid, fiber type and glycogen before and after an endurance exercise bout.** Intramyocellular lipid droplets and fiber type-I are shown in green and red stains respectively (2A). Glycogen content was measured using periodic acid schiff (PAS) stain (2B).
(TIFF)

Table S1 **Gene Assay Catalogue Numbers.**
(DOCX)
