# Supplementary figures and images for: Skeletal Muscle Perilipin 3 and Coatomer Proteins Are Increased following Exercise and Are Associated with Fat Oxidation
Source: PLoS One. 2014 Mar 14;9(3):e91675. doi: 10.1371/journal.pone.0091675 (PMC3954790; doi:10.1371/journal.pone.0091675)

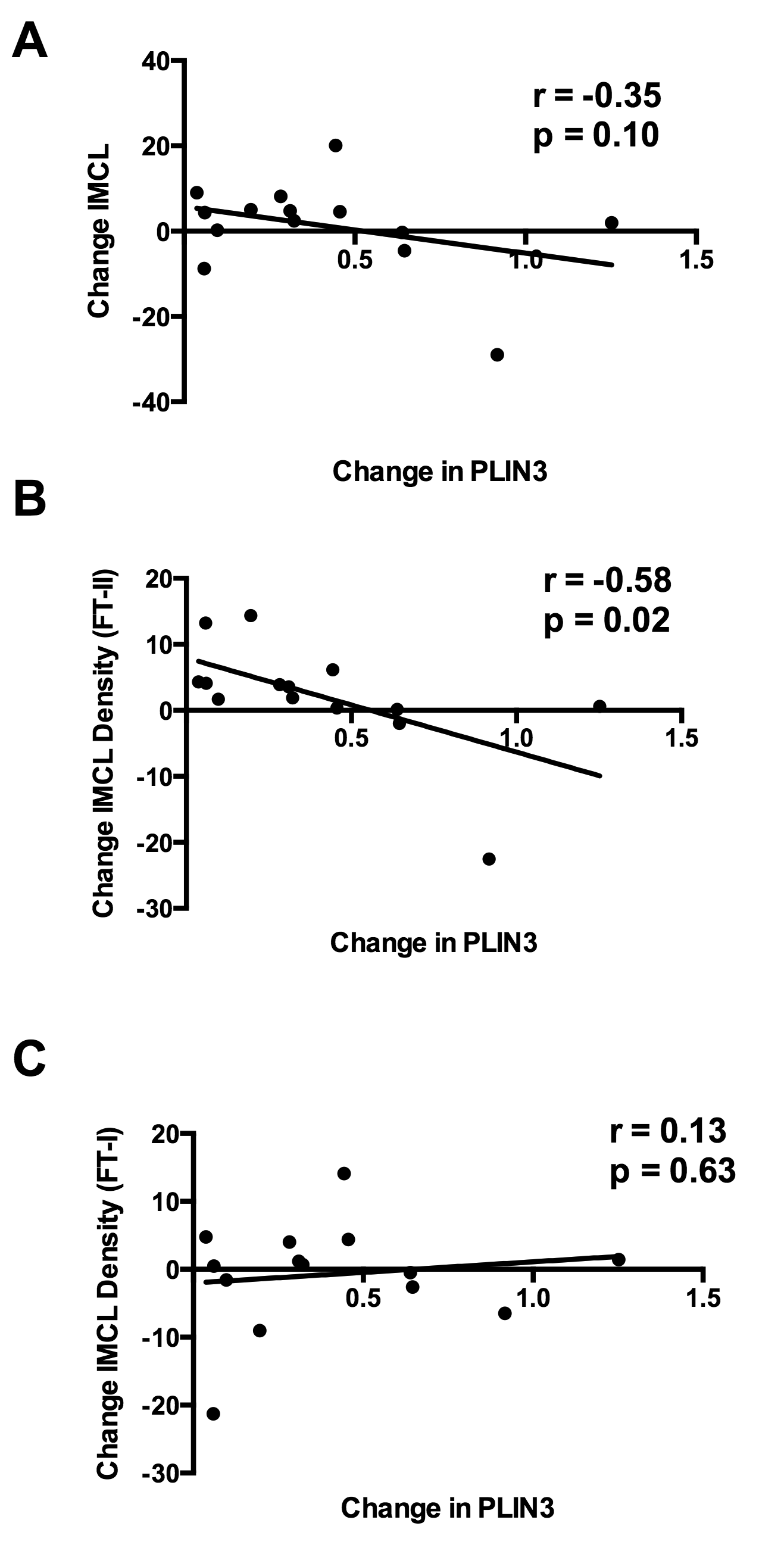

Supplement: Figure S1 — The change in PLIN3 protein content did not significantly correlate with the changes in IMCL with exercise in all participants. A) Nevertheless, for those participants who increased their PLIN3 protein content had a trend to decrease their IMCL content with exercise (p = 0.1). B) Furthermore, the change in IMCL density in type II fibers inversely related to those participants who increased their PLIN3 expression with exercise (p = 0.02). C) However, there was no significance association between the change in PLIN3 expression and the change in IMCL density in type I fibers. (TIFF) [file pone.0091675.s001.tiff]

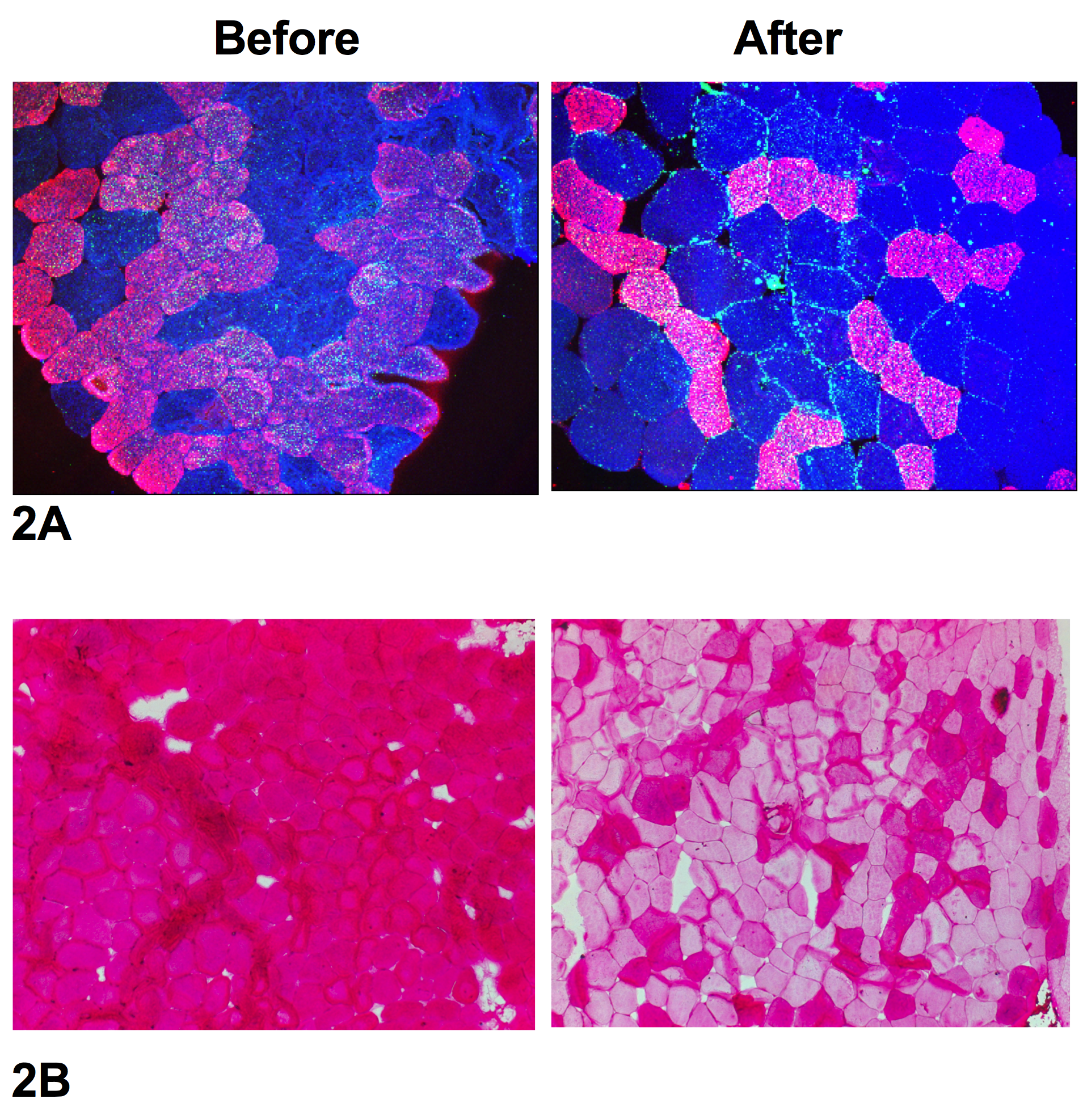

Supplement: Figure S2 — Representative images of intramyocellular lipid, fiber type and glycogen before and after an endurance exercise bout. Intramyocellular lipid droplets and fiber type-I are shown in green and red stains respectively (2A). Glycogen content was measured using periodic acid schiff (PAS) stain (2B). (TIFF) [file pone.0091675.s002.tiff]
