# Supplementary material for: Skeletal Muscle Perilipin 3 and Coatomer Proteins Are Increased following Exercise and Are Associated with Fat Oxidation
Source: PLoS One. 2014 Mar 14;9(3):e91675. doi: 10.1371/journal.pone.0091675 (PMC3954790; doi:10.1371/journal.pone.0091675)
Supplement: Table S1 — Gene Assay Catalogue Numbers. (DOCX) [file pone.0091675.s003.docx]

**Supplementary Table 1 – Gene Assay Catalogue Numbers**

| **Name** | **Symbol** | **Catalogue Number** |
| --- | --- | --- |
| Ribosomal Protein, Large Protein O | RPLPO | Hs99999902_m1 |
| Coatomer 1, beta subunit | βCOP1 | Hs00200674_m1 |
| Coatomer 2, sec23a subunit | Sec23a | Hs00197232_m1 |
| ADP-Ribosylation Factor 1 | ARF1 | Hs00796826_s1 |
| Golgi Brefelden A resistant GTPase Exchange Factor 1 | GBF1 | Hs00188327_m1 |
| ARF related peptide 1 | ARFRP1 | Hs00182389_m1 |
| PPARgamma, co-activator 1 alpha | PGC1α | Hs01016719_m1 |
| Peroxisome proliferative receptor alpha | PPARα | Hs00947538_m1 |
| Pyruvate Dehydrogenase Kinase 4 | PDK4 | Hs01037712_m1 |
| Beta-hydroxyacyl-CoA Dehydrogenase | β-HAD | Hs00193428_m1 |
